# Excess mortality during the COVID-19 pandemic in Israel, March–November 2020: when, where, and for whom?

**DOI:** 10.1186/s13584-021-00450-4

**Published:** 2021-02-26

**Authors:** Ziona Haklai, Miriam Aburbeh, Nehama Goldberger, Ethel-Sherry Gordon

**Affiliations:** Division of Health Information, Ministry of Health, Jerusalem, Israel

**Keywords:** Excess mortality, COVID-19, Lockdown

## Abstract

**Background:**

Excess all-cause mortality has been used in many countries as an estimate of mortality effects from COVID-19. What was the excess mortality in Israel in 2020 and when, where and for whom was this excess?

**Methods:**

Mortality rates between March to November 2020 for various demographic groups, cities, month and week were compared with the average rate during 2017–2019 for the same groups or periods.

**Results:**

Total mortality rates for March–November were significantly higher by 6% in 2020, than the average of 2017–2019, 14% higher among the Arab population and 5% among Jews and Others. Significantly higher monthly mortality rates were found in August, September and October by 11%, 13% and 19%, respectively, among Jews and Others, and by 19%, 64% and 40% in the Arab population.

Excess mortality was significant only at older ages, 7% higher rates at ages 65–74 and 75–84 and 8% at ages 85 and above, and greater for males than females in all ages and population groups. Interestingly, mortality rates decreased significantly among the younger population aged under 25.

The cities with most significant excess mortality were Ramla (25% higher), Bene Beraq (24%), Bat Yam (15%) and Jerusalem (8%).

**Conclusion:**

Israel has seen significant excess mortality in August–October 2020, particularly in the Arab sector. The excess mortality in March–November was statistically significant only at older ages, over 65. It is very important to protect this susceptible population from exposure and prioritize them for inoculations. Lockdowns were successful in lowering the excess mortality. The excess mortality is similar to official data on COVID-19 deaths.

**Supplementary Information:**

The online version contains supplementary material available at 10.1186/s13584-021-00450-4.

The novel severe acute respiratory syndrome coronavirus2 (SARS-CoV-2), which first emerged in December 2019 in Wuhan, China reached Israel on 27 February, 2020. By 1 December, 2020 there were 339 thousand confirmed cases of coronavirus disease 2019 (COVID-19) in Israel, more than 3.7 million tests done in an extensive network of testing laboratories, and 2863 deaths officially reported in confirmed COVID-19 cases, 88% of whom were aged 65 and above. There are likely to be other cases in persons with COVID-19 unconfirmed by laboratory tests and therefore, there may be deaths due to COVID-19 which were not attributed to the virus. Excess mortality can be used as another measure for mortality effects of COVID-19.

Analysis of excess mortality in the US [1–3] and in other countries [4, 5] has been done to try and show an accurate picture of the mortality effects of COVID-19, which may be masked, for example, in younger age groups. The OECD has published a working paper on excess mortality in 29 countries through the end of September, to help measure the direct and indirect impact of COVID-19 [6]. Excess mortality can reflect both direct deaths from COVID-19 and indirect mortality due, for example, to a reduction in seeking of care, reduction in elective surgery and procedures, pressure on the health system and ill effects of lockdown. Conversely, there might also be positive effects of the lockdowns such as fewer traffic accidents than in other years.

All deaths in Israel are reported with a notification of death form to the Ministry of Health, which issues a burial permit and passes a copy of the form to the Ministry of the Interior for issue of death certificate and update of the population registry. Notification of death forms are collated by the Central Bureau of Statistics (CBS), which codes the causes of deaths according to WHO rules, providing cause of death data available about a year and half after the end of each year, unlike complete data on the number of deaths which is available from the Ministry of the Interior within a month or two.

In this study, we explored excess mortality in Israel between March and November 2020 using data for total deaths, available by sex, age group, population group, date (month and week) and city of residence.

## Methods

We used data on all deaths in Israel provided by the Ministry of Interior. This data includes deaths of Israelis abroad for less than a year. We chose to include these deaths, to avoid bias resulting from fewer Israelis who travelled abroad in 2020. Monthly populations by population group for 2020 were obtained from the Monthly Statistical Bulletin of the CBS, and population of age groups was estimated from the total populations using the age distribution for each population group in 2019. For the city populations we used provisional estimates provided by the CBS for mid 2020.

Crude death rates between March and November 2020 for different periods and different age/sex/population groups and cities were compared with the average during 2017–2019 for the same periods and groups and the rate ratio calculated. The significance of the rate ratio was determined by a Fisher exact test. Results were considered significant when the *p*-value was ≤0.05. The analysis was done using SAS 9.4.

Although the age distribution differs considerably between different cities and population groups in Israel, it does not change significantly for each city or group over short time periods. Therefore, since we compared rates for each city and group in 2020 with their corresponding rates for average of 2017–2019, we assumed the age distribution to have remained relatively constant, and did not need age adjustment.

## Results

Between March and November 2020 there were 36,149 deaths in Israel compared to an average of 32,889 deaths between of 2017 and 2019 in these months, with an excess of 3260 deaths.

### Excess mortality by month

**Fig. 1:**
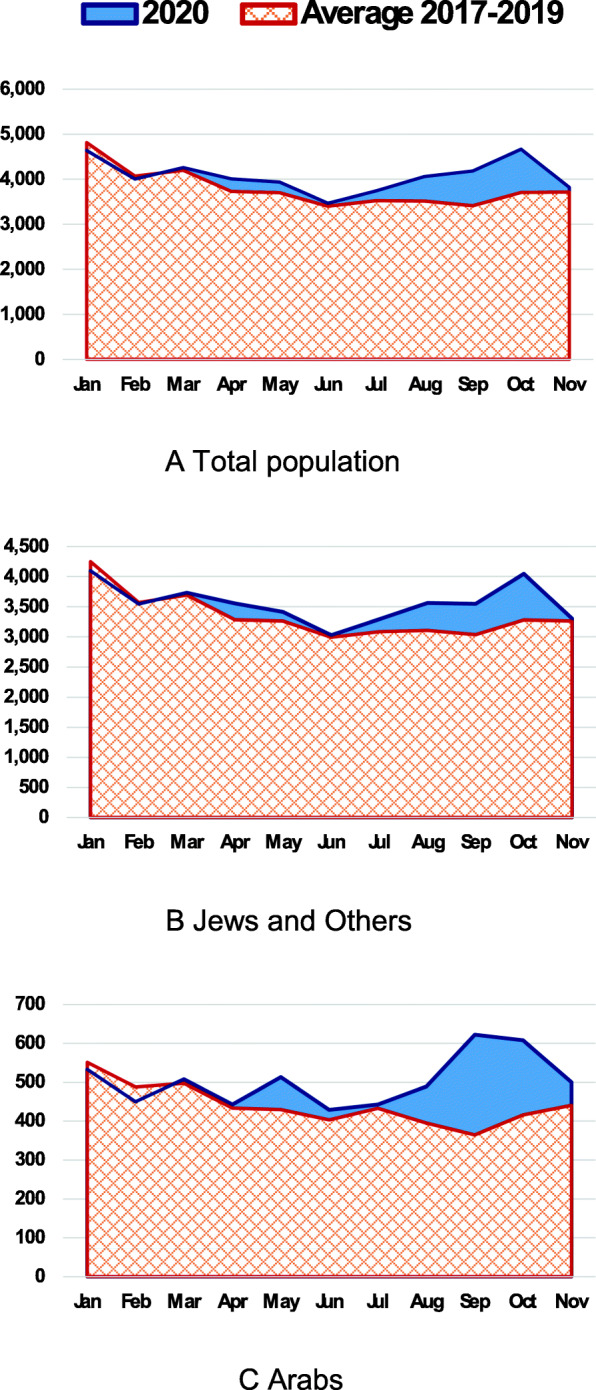
Number of deaths in 2020 compared to average in 2017–2019. **a** Total population. **b** Jews and Others. **c** Arabs

Figure 1 shows the monthly number of deaths in Israel in 2020 compared to the average in 2017–2019 for the total population, Jews and Others, and Arabs. Table 1 shows the ratio of corresponding mortality rates, and the total numbers of excess deaths and deaths in confirmed COVID-19 cases, by month. We see the excess deaths in April and May during the first wave, and a much greater excess in the second wave between August and November.

**Table 1:**
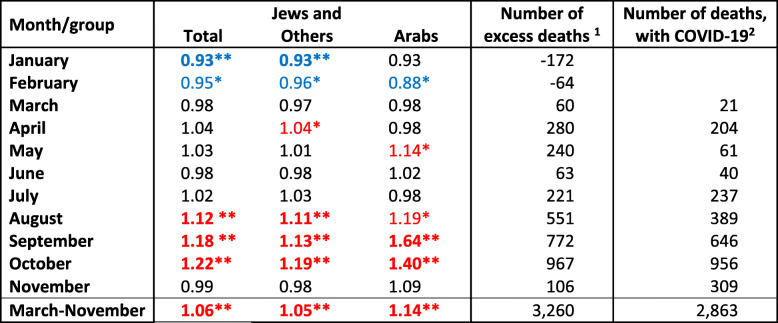
Ratio of mortality rates, 2020 compared to average of 2017–2019 by month and population group, number of excess deaths and deaths reported in confirmed COVID-19 cases

Significance of rate ratio:

**p ≤ 0.001, bold (red = high, blue = low)

*0.001 < *p* ≤ 0.05 not bold (red = high, blue = low)

^1^Difference between number of deaths in 2020 and average for 2017–2019

^2^Number of deaths of persons with confirmed COVID-19 infection

The monthly mortality rate was significantly higher in August, September and October of 2020, than the average of these months in 2017–2019, by 12%, 18% and 22%, respectively. However, in January and February, before the pandemic reached Israel, the mortality rate in 2020 was significantly less than the average in 2017–2019, 7% and 5% respectively. Arab mortality peaked in the first wave after Jews and Others, in May, when the mortality rate was significantly higher by 14%. Rates of excess mortality for the Arab population were much greater than the Jews and Others in August, September and October, 19%, 64% and 40% higher, respectively, compared to 11%, 13% and 19% among Jews and Others.

The number of excess deaths per month showed similar trends to deaths reported in confirmed COVID-19 cases. However, the excess deaths were higher in most months of the pandemic till October, but lower in November.

### Excess mortality by week

**Fig. 2:**
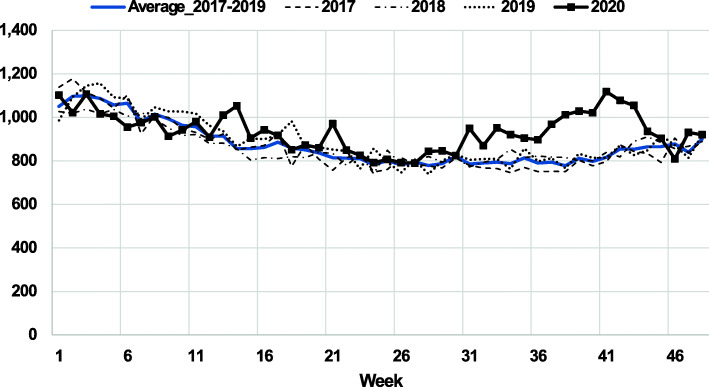
Number of deaths by week, 2020 compared to average 2017–2019, 2017, 2018, 2019

Figure 2 shows number of deaths by week in 2020 compared to the average of 2017–2019, and each of those years. The highest excess in a single week in the first wave was in the week 14 beginning 30 March 2020 (Hebrew date: 5 Nisan), with 23% excess deaths. There was a second peak in week 21 beginning 18 May, (Hebrew date: 24 Iyar), with 19% excess, corresponding to the peak in Arab deaths in May. In the second wave, excess mortality over 20% was found between week 37 and 43, peaking in week 41 beginning 5 October (Hebrew date 17 Tishrei) at 37% excess deaths.

The OECD [6] used as a comparative measure the maximum aggregate excess mortality for each country over a 10-week period in the first wave (i.e. choosing the 10-week period for which total excess mortality was highest). In Israel, in the first wave of COVID-19, the highest excess mortality over a 10-week period was 8%, for the period starting in week 13, 23 March, 2020 (Hebrew date: 27 Adar). However, in the second wave, excess deaths in the highest 10-week period was much greater at 24%, for the 10-week period starting week in 34, 17 August, (Hebrew date: 27 Av).

### Excess mortality by sex, age group and population group

Table 2 shows the ratio of mortality rates for March–November 2020 to the average of these months in 2017–2019, by sex, age groups and population group.

**Table 2:**
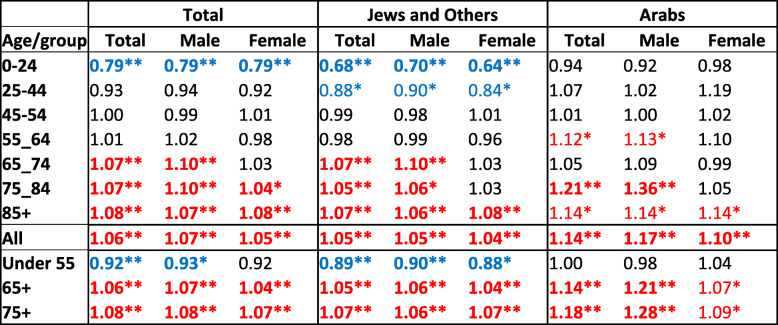
Ratio of mortality rates for March–November 2020 compared to average of 2017–2019, by age, sex and population group

Significance of rate difference:

**p ≤ 0.001, bold (red = high, blue = low)

*0.001 < *p* ≤ 0.05 not bold (red = high, blue = low)

Total mortality rates were significantly higher by 6% in 2020, by 7% for males and 5% for females, than the average of 2017–2019. The excess in mortality rates was higher for the Arab population, 14% for the total, and 17% and 10%, for males and females, respectively. Excess mortality was particularly high for older Arab males, 36% higher at ages 75–84, and 14% higher at ages 85 and above. For Arab females, mortality was only significantly higher in at age 85 and above (14%). Among Jews and Others, male mortality was significantly higher at ages 65–74 and 75–84, by 10% and 6%, respectively, while female mortality was only significantly higher at age 85 and above, 8%.compared to 6% for males of this age group.

Mortality rates were significantly higher (21%) in 2020 in Arab males aged 65 and above, and 28% higher among those aged 75 and above, compared to 6% in both age groups for Jewish and Other males. No significant excess mortality was found at younger ages, and in fact, mortality rates were significantly lower by 21% at ages 0–24, and by 32% in Jews and Others of this age in 2020 compared to 2017–2019.

### Excess mortality by city

**Fig. 3:**
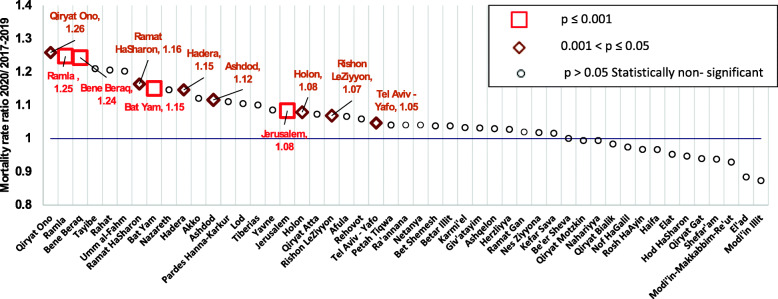
Ratio of mortality rates, March–November, 2020 compared to average 2017–2019, cites with population over 40,000

Figure 3 shows the ratio of mortality rates during March–November 2020 compared to 2017–2019 by city, for cities with population over 40,000 in 2020. Ramla and Bene Beraq had 25% and 24% higher mortality, Bat Yam (15%) and Jerusalem (8%), all highly significant (*p* value < 0.001).

Other cities with statistically significant findings (*p* value < 0.05) were Qiryat Ono (26% higher), Ramat Hasharon (16%), Hadera (15%), Ashdod (11%), Holon (8%), Rishon Le Ziyyon (7%) and Tel Aviv-Yafo (5%). It should be noted that among the major cities in Israel (population over 150,000), several did not have significantly higher mortality, including Haifa, Be’er Sheva, Petah Tiqwa, Netanya, and Ramat Gan.

Additional table 1 gives number of deaths in March–November, 2020 and 2017–2019, population in 2020, percentage aged 65 and above and 75 and above, and ratio of mortality rates for March–November, 2020 compared to average of 2017–2019, for all cities, towns and villages with populations over 10,000. Amongst Arab towns and villages, several had significantly higher mortality, including Baqa Al-Gharbiyye (32%), Qalansawe, Judeide-Maker, Deir Al-Asad, Yafi, Yirka, Fureidis and Jisr Az-Zarqa (between 42% and 57% higher mortality), although the excess mortality in larger cities like Taybe, Rahat, Umm-el-Fahm and Shefar’am was not statistically significant. The Jewish towns of Or Yehuda, Zikhron Ya’aqov and Bet She’an, also had significantly higher mortality rates in 2020, 31%, 38% and 46%, respectively. We found no city with statistically significant low mortality.

## Discussion

During the first wave of COVID-19 in Israel, between March and May, although Fig. 1 shows a small excess number of deaths, the excess in total monthly mortality rates was not statistically significant. Israel had lower excess mortality than many OECD countries. The maximum average excess mortality for a 10-week period was 8%, much lower than Spain (61%), the UK (56%), Belgium, Chile and Italy (41%–42%), and similar to other countries such as Finland, Austria and Germany.

The strict and relatively fast lockdown in April seems to have protected the population by reducing the disease rates and hence lowering excess mortality. However, during the second wave, between August and October, monthly excess mortality rates were significantly high, reaching 21% in October and 24% more cases for the highest 10-week period. In the Arab population in particular, there was very high excess mortality in September and October, 64% and 40%, respectively. We see that the September/October lockdown also succeeded in halting the increase in mortality, so that by November there was no statistically significant excess mortality.

It should be noted that we see that mortality in January and February 2020, before the COVID-19 pandemic, was lower than previous years, similar to that noted in many European countries, due to less severe and earlier seasonal diseases such as influenza in the winter of 2019/2020 [6]. It is important to compare only March to November mortality when checking for excess mortality from COVID-19, when COVID deaths began in Israel.

The total excess number of deaths between March and November 2020, compared to the average for 2017–2019, was higher than the number of deaths of COVID-19 cases, and this was particularly so in the early months of the pandemic (Table 1). This could be partially due to inadequate availability of testing at the beginning of the pandemic. Other excess deaths could be due to indirect results of COVID-19, such as less routine medical services and deleterious effects of lockdown. A more correct analysis of the deaths actually due to COVID-19 cannot be done until the complete coded cause of death file for 2020 is available, but excess mortality gives a good indication.

We found significantly lower mortality rates in 2020 than the average of 2017–2019 in the younger population aged 0–44. The protective effects of lockdown might explain this lower rate from causes of deaths such as accidents, which rank among the primary leading causes of death at this age [7].

Excess mortality was significant and high among the older ages, over 75, and was higher in particular among Arab males. These results highlight the importance of people in these age groups taking strict precautions to avoid the disease, and of the inoculations that are now being administered to them.

We saw significant geographical differences in excess mortality. Twenty-two towns with population over 10,000 had statistically significant excess mortality, including eight from the Arab sector, and the cities with highest significance were Ramla, Bene Beraq, Bat Yam and Jerusalem.

Before the second wave of disease began, the advisory committee on COVID-19 to the Centre for National Security analyzed the factors likely to contribute to high infection rates such as crowded living conditions, large families, a large proportion of 15–29 year olds, low socio-economic status and frequent use of public transport. The list of cities they concluded were likely to be at highest risk had mostly large Arab and Haredi populations [8]. The significant excess mortality in Bene Beraq, a Haredi city, and Jerusalem, with both a large Haredi and Arab population supported this prediction, but the higher mortality rates in Ramat Hasharon and Rishon LeZiyyon were not predicted.

The large majority of COVID-19 mortality is at aged 65 and above, and therefore we might expect the proportion of population that age to be an important factor predicting excess mortality, unlike the proportion of younger population, which predicts high infection rates. This was demonstrated in the Haredi cities of Betar Illit, El’ad and Modi’in Illit, which had high infection rates [9], but did not have significant excess mortality, since they have young populations,1.6% or less are aged 65 and above in these cities, as compared to Bene Bereq with 7 % elderly (additional table 1).

Bat Yam also has a relatively high proportion aged 65 and over, 22% as does Ramat Hasharon (19%), Holon (18%) and Rishon LeZiyyon (17%) which may contribute to their high excess mortality. However, other cities such as Haifa, Ramat Gan and Netanya, also have relatively large older populations (18%–20%), and Petah Tiqwa and Be’er Sheva are only slightly less (16%), but nevertheless did not have significant excess mortality. Low socio-economic status may contribute to high excess mortality, such as in Ramla. There have to be other important contributing factors such as the municipal reaction to the situation and their co-operation with police and health authorities. It should also be noted that a mild excess mortality in large cities could be statistically significant, such as the 5% excess in Tel Aviv-Yafo.

The Arab sector had particularly high significant excess mortality overall, also found in eight towns and villages, despite their young age (only 3%–6% over 65), and this was found more for males than females. The other factors which lead to high infection rates followed by high mortality appear more important, such as low socio-economic status and frequent social interactions. In addition, the family centered Arab lifestyle, with multi-generational family homes may have led to the elderly being more exposed and vulnerable.

## Limitation

This study is based on total mortality data in Israel available at time of writing, which is assumed to be complete through November 2020. Minor adjustments could occur to the numbers of deaths, but are unlikely to change the results significantly.

The populations used to calculate rates was also estimated from available data, and may be subject to correction.

## Conclusion

Israel has seen significant excess mortality in August–October 2020, particularly in the Arab sector. The excess mortality in March–November was statistically significant only at older ages, over 65. It is very important to protect this susceptible population from exposure and prioritize them for inoculations. Lockdowns were successful in lowering the excess mortality. The excess mortality is similar to official data on COVID-19 deaths.

## Supplementary Information

**Additional file 1: Additional table 1.** Mortality data for cities with population over 10,000 in 2020, March–November, 2020 compared to 2017–2019.

## Data Availability

Data on mortality and causes of death in Israel are available from the WHO mortality database (last updated December 2019). Data on COVID-19 tests, cases and deaths are available from the Israeli government databases site [9].

## Abbreviations

COVID-19: Corona virus disease 2019
CBS: Central Bureau of Statistics
WHO: World Health Organization
